# Transitioning from multiport to uniport robot-assisted thoracic surgery: a propensity score–weighted evaluation of safety and feasibility

**DOI:** 10.3389/fsurg.2026.1842961

**Published:** 2026-07-24

**Authors:** In Ha Kim, Jae Kwang Yun

**Affiliations:** Department of Thoracic and Cardiovascular Surgery, Asan Medical Center, University of Ulsan College of Medicine, Seoul, Republic of Korea

**Keywords:** IPTW, lung cancer, multiport, robotic-assisted thoracic surgery, uniport, uRATS

## Abstract

**Background:**

The present study assessed the safety and feasibility of uniport robot-assisted thoracic surgery (RATS) during a structured transition from conventional multiport to uniport RATS.

**Methods:**

Patients undergoing RATS pulmonary resection for non–small cell lung cancer between June 2022 and December 2025 were included in this retrospective analysis. Perioperative outcomes were evaluated according to surgical approach (uniport vs. multiport). To address potential baseline differences between the groups, inverse probability of treatment weighting (IPTW) based on propensity scores was employed.

**Results:**

The study cohort comprised 154 patients, with all operations performed by a single surgeon. Of these, 53 (34.4%) underwent uniport RATS and 101 (65.6%) underwent multiport RATS. After IPTW adjustment, baseline variables were adequately balanced across the groups. Complete resection was achieved in all cases, with one conversion in the multiport group. The surgical extent, histologic type, and lymph node assessment were comparable between groups. In our series, operative duration was shorter among patients undergoing uniport RATS both before (131.5 ± 23.4 vs. 145.8 ± 43.9 min, *P* = 0.037) and after IPTW (130.1 ± 23.0 vs. 147.3 ± 46.2 min, *P* = 0.004). Duration of chest drainage, postoperative hospital day, and complication rates were similar between groups. Patients in the uniport RATS group reported lower postoperative pain scores on postoperative days 0–2, and this difference remained significant in both the crude and IPTW-weighted analyses (all *P* < 0.05).

**Conclusions:**

Within a structured transition pathway, uniport RATS achieved perioperative outcomes and early oncologic feasibility comparable to those of multiport RATS, supporting its safety and technical applicability.

## 1 Introduction

Minimally invasive techniques in thoracic surgery have evolved substantially since the introduction of multiport video-assisted thoracoscopic surgery (VATS) lobectomy in 1992 (1). Over time, surgeons with sufficient technical proficiency have sought to further minimize invasiveness by reducing the number of ports while maintaining satisfactory surgical outcomes (2, 3). This effort led to the first report of uniport VATS pulmonary resection in 2010 (4). Subsequently, several institutions have adopted and standardized uniport VATS as a viable approach (5, 6).

Another development in minimally invasive thoracic surgery is robot-assisted thoracic surgery (RATS), with the first multiport pulmonary resection reported in 2002 (7). RATS offers technical advantages, including three-dimensional visualization and improved instrument dexterity, which have promoted its adoption in thoracic surgery (8, 9). By 2020, RATS accounted for over half of pulmonary resections performed in the United States (10). However, contrary to the expectation of a less invasive approach, conventional multiport RATS typically requires four or more ports, making it more invasive than uniport VATS. Similar to the advancements in VATS, some surgeons have explored reducing the number of ports in RATS while maintaining acceptable surgical outcomes. Recent reports have documented successful outcomes with three-port and two-port RATS (11, 12), and pioneering surgeons have described their early experiences with uniport RATS pulmonary resection (13–15). However, uniport RATS has not yet been widely adopted, and its safety and feasibility have not been fully validated. Moreover, most published reports on uniport RATS have been descriptive in nature, with limited direct comparisons with the multiport approach, which makes it challenging to determine the safety of this technique.

Therefore, the present study evaluated the safety and feasibility of uniport RATS during a structured transition from conventional multiport to uniport RATS by comparing perioperative outcomes between the two approaches.

## 2 Materials and method

### 2.1 Patients

We performed a retrospective single-center study at Asan Medical Center. Patients with non-small cell lung cancer who underwent RATS pulmonary resection between June 2022 and December 2025 were included, while those who received neoadjuvant treatment were excluded. Preoperative evaluation included histologic confirmation, chest computed tomography (CT), brain magnetic resonance imaging, positron emission tomography-CT, pulmonary function tests, and laboratory examinations. When nodal assessment was required, endobronchial ultrasound was utilized. For patients without a preoperative histologic diagnosis, an intraoperative wedge resection with frozen biopsy was initially performed, followed by the main pulmonary procedures. Demographic, clinicopathological, and perioperative data were extracted from a prospectively collected database. A single surgeon performed all operations in this series. Initially, a four-port, four-arm approach was employed; however, efforts were made to reduce the number of ports and robotic arms used. This transition progressed through a two-port, three-arm approach and ultimately evolved into a uniport, three-arm approach. Currently, the uniport, three-arm approach is the author's standard procedure. However, in cases where severe adhesions or severe anthracotic lymph nodes (LNs) are anticipated, or in patients who have undergone neoadjuvant treatment, a multiport approach is selectively adopted.

### 2.2 Surgical technique

#### 2.2.1 Multiport

**Figure 1:**
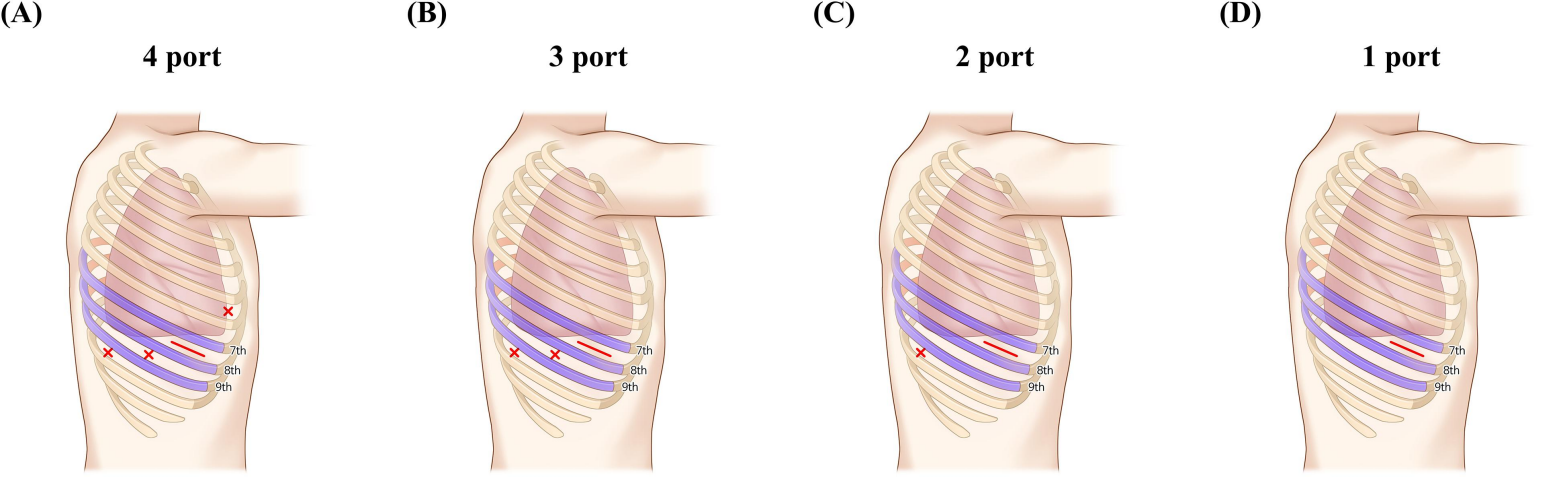
Port placements of multiport and uniport robot-assisted thoracic surgery. **(A)** 4-port, **(B)** 3-port, **(C)** 2-port, **(D)** 1-port.

Following induction of general anesthesia, single-lung ventilation was established. Patients were placed in the lateral decubitus position with the table flexed at the level of the xiphoid process. The operations were performed using the da Vinci Xi or da Vinci X robotic system (Intuitive Surgical Inc., Sunnyvale, CA, USA). The robotic platform was docked from the patient's dorsal side. The main port was made at the 7th intercostal space (ICS), spanning the anterior (AXL) to the middle axillary line (MXL), with a size of 3–4 cm, and a wound retractor (Applied Medical, Rancho Santa Margarita, CA, USA) was placed. Additional ports were established at the 5th ICS along the AXL, the 8th ICS along the MXL, and the 9th or 10th ICS along the posterior axillary line (PXL), with an 8 mm trocar used for robotic arm installation (Figure 1A). The vessel sealer was introduced at the 5th ICS port and the robotic camera at the 8th ICS port. The operator typically utilized the remaining ports, using a Maryland Bipolar Forceps in the right hand and Cadiere Grasper in the left hand to perform the procedure. A 45 mm robotic SureForm stapler (Intuitive Surgical, Sunnyvale, CA, USA), which has a straight stapler profile, was utilized to divide the vascular and bronchial structures. A 12 mm port was placed at the main wound to accommodate the robotic stapler. After dissection around the pulmonary vessels and bronchus, a 15 cm Penrose drain was placed beneath the structures. Subsequently, the tip of the robotic stapler was inserted into the drain to guide the stapler safely, facilitating secure division. Small pulmonary arteries were divided using robotic Hem-o-lok clips. Gauze ball was used to maintain a clear surgical field and for lung retraction using a vessel sealer. The assistant surgeon, positioned on the patient's ventral side, performed suction, tissue retraction, gauze changes, and extraction of the excised LNs through the main wound. Additionally, as CO_2_ insufflation was not employed, air-tightening trocars were not necessary. A chest tube was placed through the 8th ICS middle axillary port.

After several successful cases of robotic surgery utilizing a four-port, four-arm approach, efforts were made to reduce the number of ports used. A three-port, three-arm approach was attempted by omitting the port traditionally placed at the 5th ICS along the AXL (Figure 1B). Further advancements led to the implementation of a two-port, three-arm robotic surgery technique, wherein the main incision was made at the 7th ICS between the AXL and MXL, and an 8 mm port was placed at the 9th ICS along the PXL (Figure 1C). Two 8 mm ports were inserted at both ends of the main wound, and a robotic camera was installed at the posterior side of the main wound. In most cases, the left arm was equipped with a Cadiere grasper, and the right arm was equipped with Maryland bipolar forceps. When a robotic stapler was required, the 8 mm port docked into the main wound was replaced with a 12 mm port.

#### 2.2.2 Uniport

**Figure 2:**
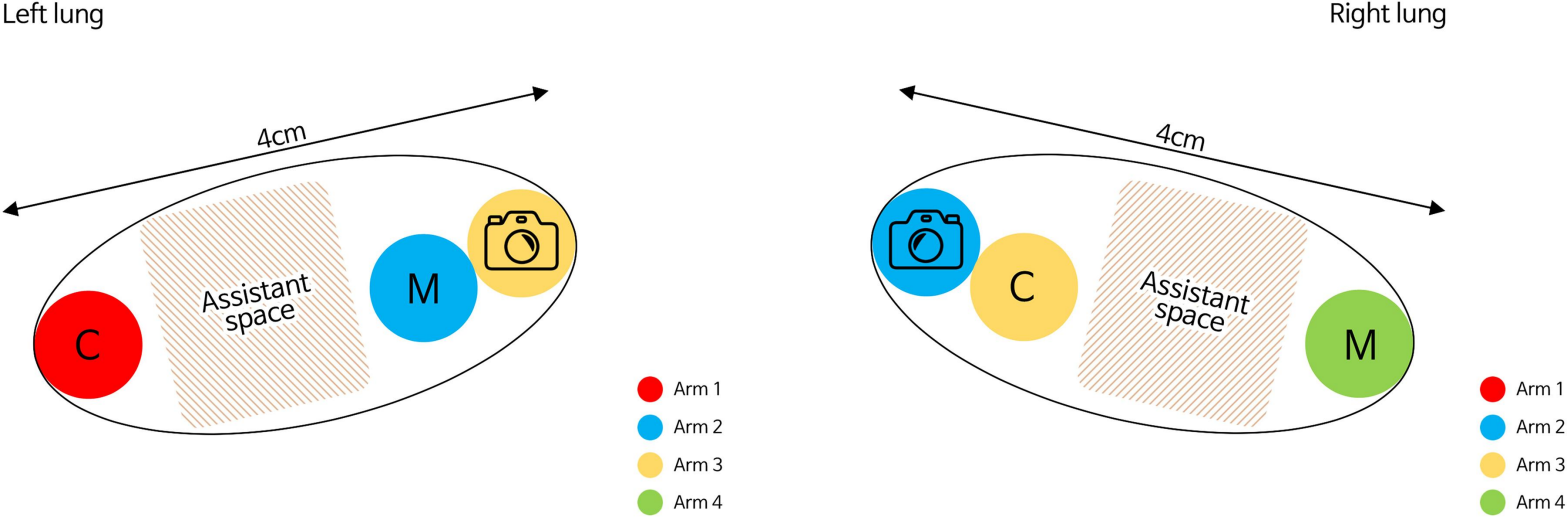
Schematic diagram of robotic arm installation in uniport robot-assisted thoracic surgery.

A single incision, with a size of 4 cm, was made at the 7th ICS between the AXL and MXL, through which all three robotic arms were introduced in parallel (Figure 1D). The robotic camera was placed on the patient's dorsal side, and the remaining two ports were placed at the maximum distance from each other within the main wound (Figure 2). During the procedure, the operator predominantly used a Maryland bipolar forceps in the right arm and Cadiere grasper in the left arm. A 45 mm SureForm robotic stapler was installed on the appropriate arm as needed, depending on the surgical situation. Systematic hilar and mediastinal LN dissection was routinely performed during anatomic lung resection, with a consistent nodal assessment strategy applied across both multiport and uniport RATS.

### 2.3 Pain control & postoperative management

After general anesthesia, a paravertebral block was performed at the T4 and T6 levels using 20 mL 0.25% ropivacaine. An intercostal nerve block was administered at the intercostal levels where port placement had been performed, using 20 mL of 0.75% ropivacaine, prior to skin closure. A patient-controlled analgesia infusion pump was started before surgery completion, and oral analgesics were administered following the resumption of oral intake. Postoperative pain was evaluated using the Numeric Rating Scale at 8 h intervals, with the highest pain intensity recorded each day. All patients were extubated after surgery, and early ambulation and lung care were implemented. Chest tube removal was considered when daily drainage was ≤4 mL/kg in the absence of air leakage.

### 2.4 Statistical analysis

Categorical variables are summarized as counts with percentages and were compared using the chi-square or Fisher's exact test. Continuous variables are reported as means and standard deviations and were evaluated using Student's t test or the Wilcoxon rank-sum test. To adjust for potential baseline imbalances between the multiport and uniport groups, inverse probability of treatment weighting (IPTW) based on propensity scores (PS) was applied. The PS, representing the probability of undergoing uniport RATS, was estimated using a multivariable logistic regression model including age, sex, body mass index (BMI), smoking history, number of comorbidities, Eastern Cooperative Oncology Group (ECOG) performance status, forced expiratory volume in 1 s (FEV1), diffusing capacity for carbon monoxide (DLCO), clinical tumor size, and tumor laterality. IPTW weights were calculated as 1/PS for the uniport group and 1/(1−PS) for the multiport group. To reduce the influence of extreme weights due to potential model misspecification, weights were trimmed to a range of 0.2–5. Balance between groups was assessed using standardized mean differences (SMDs). An SMD ≤ 0.1 was considered to indicate optimal balance, and ≤0.2 acceptable balance. Operation time was defined as the duration from incision to skin closure. Conversion was defined as additional port insertion or open thoracotomy. Postoperative complications were classified using the Clavien–Dindo classification (16). The harvested LN stations and harvested LNs refer to the number of evaluated LN stations and the number of dissected LN fragments, respectively. To explore the potential impact of the learning curve on operative efficiency, cumulative sum (CUSUM) analyses of operative time were performed separately for the multiport and uniport cohorts. Cases were arranged chronologically according to the order in which they were performed. The change point was defined as the case at which the CUSUM curve reached its maximum value, representing the transition from the initial learning phase to a more stable level of operative performance. All statistical analyses were performed using R version 4.5.1 (The R Foundation for Statistical Computing, Vienna, Austria). Two-sided tests were conducted for all analyses, with statistical significance set at *P* < 0.05.

## 3 Results

Overall, 154 patients underwent RATS pulmonary resection between June 2022 and December 2025. Among them, 101 (65.6%) underwent multiport RATS, including four-port (*n* = 75), three-port (*n* = 15), and two-port (*n* = 11) approaches, while the remaining 53 (34.4%) underwent uniport RATS. The cohort size remained unchanged after IPTW adjustment. Table 1 summarizes the baseline characteristics of the study population. Before adjustment, there were no meaningful differences between the groups regarding age, sex, BMI, smoking history, number of comorbidities, ECOG performance status, FEV1, DLCO, clinical tumor size, laterality, or tumor location. After IPTW adjustment, adequate covariate balance was achieved with standardized mean differences <0.2 for all variables except tumor location.

**Table 1:** Baseline characteristics of the included patients who underwent multiport and uniport robot-assisted thoracic surgery.

Variables	Overall cohort				IPTW-adjusted cohort			
	Multiport (*N* = 101)	Uniport (*N* = 53)	*P* value	SMD	Multiport (*N* = 101)	Uniport (*N* = 53)	*P* value	SMD
Age (years)	63.4 ± 8.7	64.6 ± 6.8	0.371	0.158	64.0 ± 8.2	64.6 ± 6.6	0.621	0.082
Sex (male)	52 (51.5%)	25 (47.2%)	0.734	0.086	50 (49.5%)	25 (47.2%)	0.795	0.048
BMI (kg/m^2^)	24.8 ± 3.4	24.3 ± 3.3	0.415	0.139	24.6 ± 3.4	24.5 ± 3.4	0.898	0.024
Smoking history	44 (43.6%)	18 (34.0%)	0.326	0.198	40 (39.6%)	21 (39.6%)	0.972	0.007
Numbers of comorbidities			0.616	0.171			0.801	0.122
0–1	71 (70.3%)	41 (77.4%)			75 (74.3%)	40 (75.5%)		
2	21 (20.8%)	9 (17.0%)			18 (17.8%)	8 (15.1%)		
≥3	9 (8.9%)	3 (5.7%)			8 (7.9%)	5 (9.4%)		
ECOG performance status			0.104	0.320			0.821	0.046
0	68 (67.3%)	43 (81.1%)			73 (72.3%)	37 (69.8%)		
1	33 (32.7%)	10 (18.9%)			28 (27.7%)	16 (30.2%)		
FEV1 (%)	90.6 ± 13.9	89.5 ± 14.1	0.651	0.077	90.3 ± 13.8	92.1 ± 15.5	0.575	0.119
DLCO (%)	87.5 ± 14.7	91.4 ± 15.7	0.128	0.257	89.1 ± 15.3	89.3 ± 15.8	0.939	0.014
Clinical tumor size (mm)	28.0 ± 11.1	26.6 ± 11.8	0.472	0.121	27.3 ± 11.1	27.8 ± 13.8	0.862	0.036
Laterality			0.272	0.218			0.506	0.121
Right	56 (55.4%)	35 (66.0%)			61 (60.4%)	35 (66.0%)		
Left	45 (44.6%)	18 (34.0%)			40 (39.6%)	18 (34.0%)		
Location			0.091	0.490			0.065	0.554
RUL	33 (32.7%)	16 (30.2%)			37 (36.6%)	14 (26.4%)		
RML	6 (5.9%)	8 (15.1%)			6 (5.9%)	9 (17.0%)		
RLL	17 (16.8%)	11 (20.8%)			17 (16.8%)	12 (22.6%)		
LUL	34 (33.7%)	9 (17.0%)			31 (30.7%)	9 (17.0%)		
LLL	11(10.9%)	9(17.0%)			10(9.9%)	9(17.0%)		

Variables are summarized as counts (%) or mean ± standard deviation. IPTW, inverse probability of treatment weighting; SMD, standardized mean difference; BMI, body mass index; ECOG, eastern cooperative oncology group; FEV1, forced expiratory volume in one second; DLCO, diffusing capacity of the lung for carbon monoxide; RUL, right upper lobe; RML, right middle lobe; RLL, right lower lobe; LUL, left upper lobe; LLL, left lower lobe.

**Figure 3:**
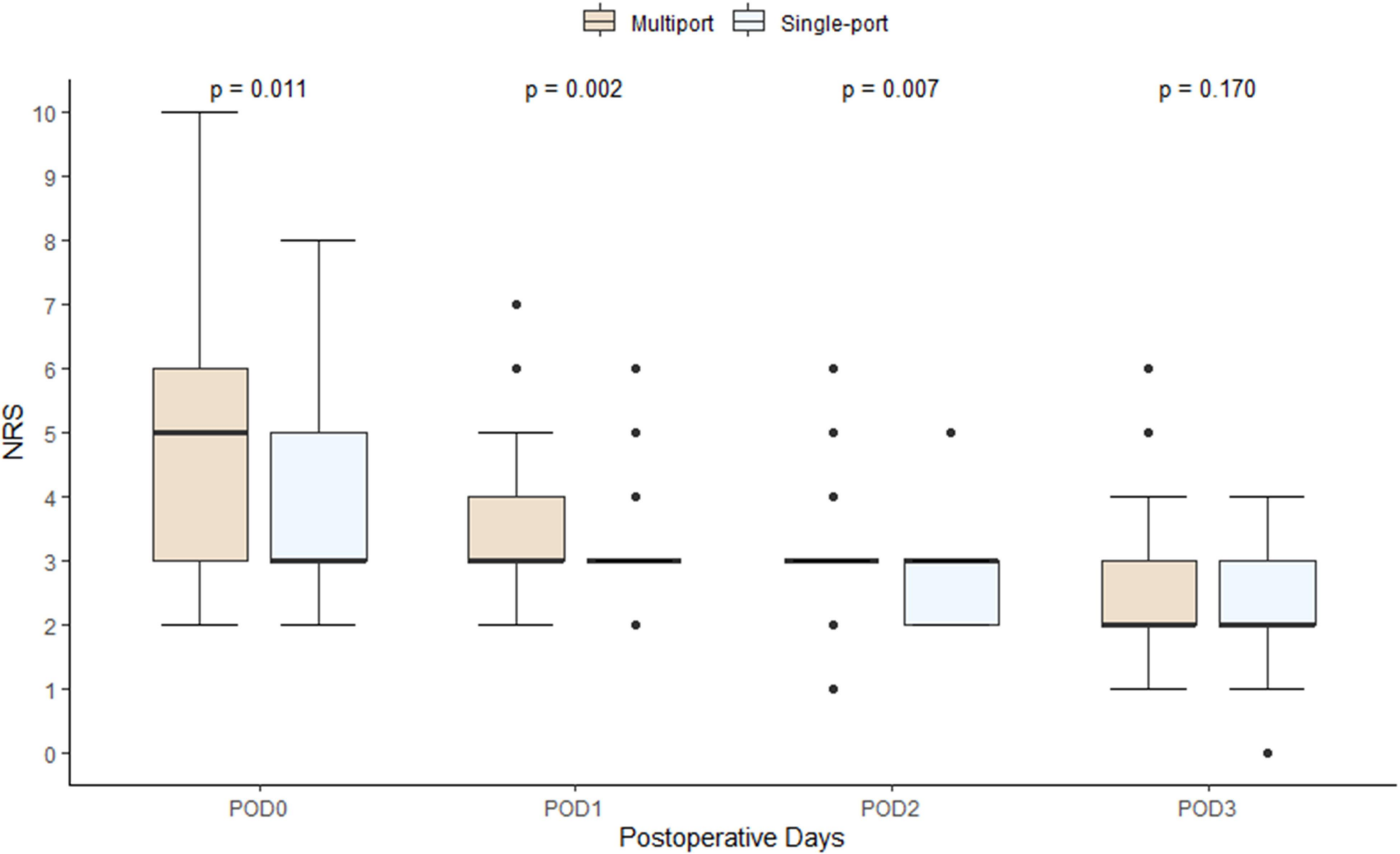
Postoperative pain scores of uniport versus multiport robot-assisted thoracic surgery.

Perioperative outcomes before and after IPTW adjustment are presented in Table 2. Complete resection was performed in all cases, although one conversion occurred in the multiport RATS group. No significant differences were observed between groups in surgical extent, histologic type, number of harvested LNs, number of LN stations, or pathologic tumor size in either the unadjusted or IPTW-adjusted analyses. Postoperative outcomes, including Clavien–Dindo complications, duration of chest tube, and postoperative hospital day, were comparable between groups. Operation time was shorter in the uniport RATS group both before (145.8 ± 43.9 vs. 131.5 ± 23.4 min, *P* = 0.009) and after IPTW adjustment (147.3 ± 46.2 vs. 130.1 ± 23.0 min, *P* = 0.004). Patients in the uniport RATS group reported lower postoperative pain scores on postoperative days 0–2, and this difference remained significant in both the crude and IPTW-weighted analyses (all *P* < 0.05) (Figure 3).

**Table 2:** Perioperative outcomes of included patients who underwent multiport and uniport robot-assisted thoracic surgery.

Variables	Overall cohort			IPTW-adjusted cohort		
	Multiport (*N* = 101)	Uniport (*N* = 53)	*P* value	Multiport (*N* = 101)	Uniport (*N* = 53)	*P* value
Operation time (min)	145.8 ± 43.9	131.5 ± 23.4	0.009	147.3 ± 46.2	130.1 ± 23.0	0.004
Complete resection	101 (100.0%)	53 (100.0%)	NA	101 (100.0%)	53 (100.0%)	NA
Conversion	1 (1.0%)	0 (0.0%)	0.99	1 (1.0%)	0 (0.0%)	0.99
Surgical extent			0.142			0.068
≥Lobectomy	76 (75.2%)	46 (86.8%)		84 (83.2%)	46 (86.8%)	
Sublobar resection	25 (24.8%)	7 (13.2%)		27 (26.7%)	7 (13.2%)	
Histologic type			0.178			0.154
Adenocarcinoma	84 (83.2%)	49 (92.5%)		86 (85.1%)	49 (92.5%)	
Non-adenocarcinoma	17 (16.8%)	4 (7.5%)		15 (14.9%)	4 (7.5%)	
Harvested LNs	25.2 ± 10.4	24.8 ± 9.2	0.829	25.0 ± 10.5	24.2 ± 9.2	0.671
Harvested LN stations	7.8 ± 1.8	7.3 ± 1.5	0.101	7.7 ± 1.9	7.3 ± 1.5	0.187
Pathologic tumor size (mm)	27.7 ± 12.0	25.6 ± 11.9	0.296	27.0 ± 11.9	26.4 ± 14.0	0.840
Clavien-Dindo complication			0.481			0.517
0	88 (87.1%)	45 (84.9%)		89 (88.1%)	46 (86.8%)	
I	3 (3.0%)	1 (1.9%)		3 (3.0%)	1 (1.9%)	
II	3 (3.0%)	0 (0.0%)		2 (2.0%)	0 (0.0%)	
IIIa	4 (4.0%)	5 (9.4%)		4 (4.0%)	5 (9.4%)	
IIIb	2 (2.0%)	2 (3.8%)		2 (2.0%)	1 (1.9%)	
IV	1 (1.0%)	0 (0.0%)		1 (1.0%)	0 (0.0%)	
Chest drainage (days)	3.3 ± 2.3	3.5 ± 2.0	0.601	3.3 ± 2.2	3.5 ± 1.9	0.657
Hospital stay (days)	4.6 ± 3.2	4.5 ± 1.9	0.753	4.6 ± 2.9	4.1 ± 1.8	0.709
NRS pain scale						
POD 0	4.8 ± 2.0	4.0 ± 1.6	0.012	4.8 ± 2.0	4.1 ± 1.6	0.027
POD 1	3.5 ± 1.1	3.0 ± 0.8	0.002	3.5 ± 1.0	3.0 ± 0.8	0.001
POD 2	3.0 ± 0.8	2.6 ± 0.7	0.018	2.9 ± 0.8	2.6 ± 0.6	0.006
POD 3	2.4 ± 0.8	2.2 ± 0.8	0.091	2.4 ± 0.7	2.1 ± 0.8	0.075

Variables are summarized as counts (%) or mean ± standard deviation. IPTW, inverse probability of treatment weighting; LN, lymph node; NRS, numeric rating scale; POD, postoperative day.

**Figure 4:**
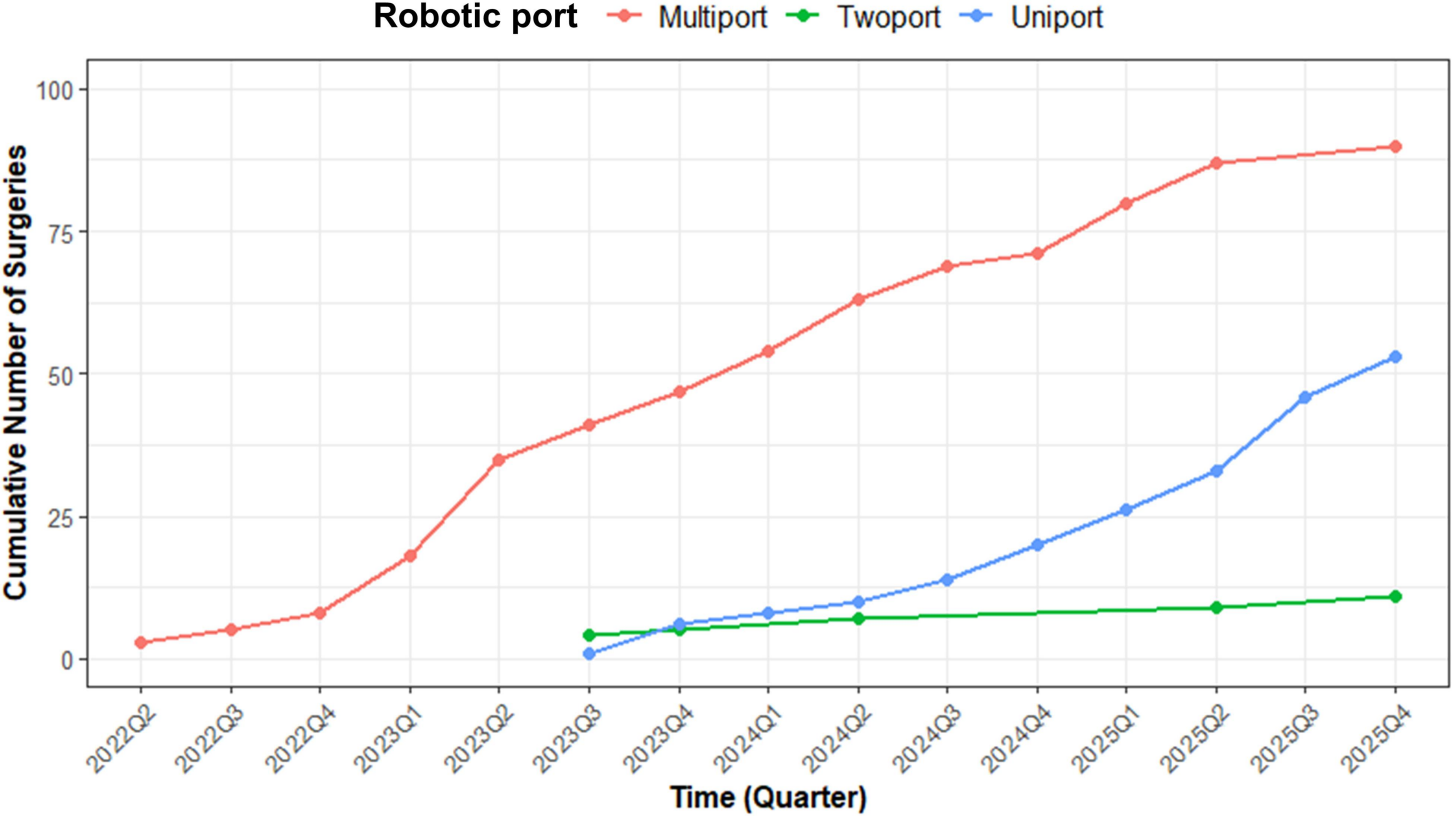
Cumulative number of cases for each port in robot-assisted thoracic surgery during the study period.

A stepwise reduction in the number of ports was achieved, with two-port RATS introduced after completing 36 multiport (three- or four-port) RATS cases. Following the successful execution of two 2-port RATS procedures, a uniport RATS was performed. Over time, the proportion of uniport RATS procedures progressively increased (Figure 4).

**Figure 5:**
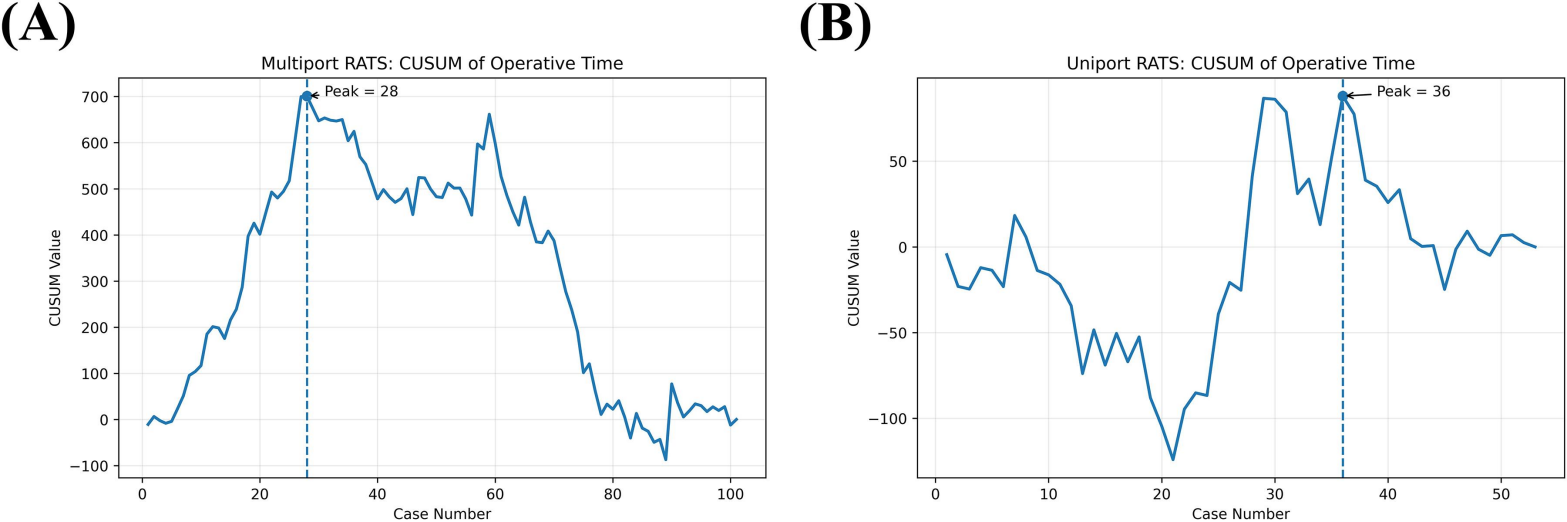
Learning-curve assessment using cumulative sum (CUSUM) analysis of operative time. CUSUM curves for operative time are presented for the multiport **(A)** and uniport **(B)** cohorts.

CUSUM analysis of operative time was performed separately for the multiport and uniport cohorts. The CUSUM curve reached its maximum value at the 28th case in the multiport cohort and at the 36th case in the uniport cohort (Figure 5).

## 4 Discussion

This single-center, single-surgeon retrospective study compared the perioperative outcomes of uniport and multiport RATS pulmonary resection in patients with lung cancer. Perioperative outcomes were comparable between the two techniques without surgery-related mortality, and similar findings were observed after IPTW adjustment. Although survival analysis was not performed, both groups demonstrated adequate lymph node assessment, supporting the technical adequacy of oncologic resection and LN assessment. Notably, uniport RATS showed an advantage in postoperative pain control.

RATS has become an increasingly popular minimally invasive technique in thoracic surgery owing to its advantages (10). Although VATS has been standardized as three-port or uniport procedure, conventional RATS typically requires four or more ports, making it more invasive. Nonetheless, technological advancements and dedicated efforts by surgeons have led to stepwise reductions in the number of ports, with recent reports of uniport RATS for lung cancer (13, 17). The primary concern remains the safety. A European multicenter study comparing uniport and multiport RATS in 101 patients observed comparable postoperative outcomes, including ICU stay, complications, and mortality (18). Other studies comparing uniport RATS and uniport VATS have also reported similar perioperative outcomes (17, 19). Our findings further support the feasibility and safety of uniport RATS, including the initial cases, when implemented within a structured transition pathway and in experienced hands. These consistent findings suggest that reducing the number of ports does not compromise the technical advantages of the robotic platform. Our study further demonstrates the feasibility of achieving these outcomes through a structured transition strategy.

The primary contribution of this study lies not in a simple comparison between uniport and multiport RATS, but in its evaluation of a structured transition from conventional multiport to uniport RATS. The sequential reduction from a four-port, four-arm approach to three-port, two-port, and ultimately uniport three-arm RATS allowed the surgeon and operating team to adapt progressively to more constrained instrument alignment, arm spacing, stapler insertion, and assistant access while retaining familiarity with the robotic platform. This stepwise strategy may provide a practical framework for introducing reduced-port RATS. However, the transition sequence and case numbers observed in this single-surgeon series should not be regarded as universal proficiency thresholds.

All procedures in this study were performed by a single surgeon, and the study included patients from the surgeon's initial experience with RATS. With increasing experience, operative time decreased and the number of ports was successfully reduced. RATS has a relatively short learning period, allowing surgeons to achieve technical proficiency after a limited number of cases (20, 21). In the present study, transition to the uniport approach was successfully achieved after approximately 40 multiport cases, suggesting that adoption of uniport RATS is feasible after acquisition of limited robotic experience. Similarly, a Japanese study introduced uniport RATS after only 6 multiport cases and reported favorable outcomes during the initial 25 uniport procedures (17), suggesting that uniport RATS is reproducible with prior experience with the robotic platform and uniport VATS. Given that uniport RATS was introduced after a stepwise transition through the multiport approach, technical skills acquired during the multiport phase, including robotic console handling, hilar dissection, and robotic stapling, were likely transferable and may have contributed to the shorter operative time observed in the uniport group. Consistent with this interpretation, CUSUM analysis identified change points at the 28th and 36th cases in the multiport and uniport cohorts, respectively, indicating learning-curve effects in both approaches. Accordingly, the shorter operative time observed in the uniport group was likely attributable not only to increasing surgical experience but also to the transfer of technical skills acquired during the preceding multiport phase.

During the study period, our institution followed a consistent pain control strategy, as described in the Methods section. Pain scores on postoperative days 0, 1, and 2 were lower in the uniport RATS group, consistent with previous studies (22). A recent Japanese prospective multicenter study analyzed postoperative pain in patients undergoing RATS and VATS (23) and observed no significant difference in pain levels between the two approaches; however, factors such as the number of ports, largest wound size, and tumor location were associated with postoperative pain. Although not evaluated in this study, patient satisfaction with cosmetic outcomes improved because of the single incision, a finding supported by previous studies (24).

An important consideration in the surgical treatment of lung cancer is securing adequate oncologic outcomes. Reducing the number of ports to achieve a less invasive approach is a promising advancement; however, acceptable oncological outcomes must be ensured. Owing to its inherent characteristics, RATS has been reported to be superior to VATS for LN (25, 26). Moreover, a European study comparing uniport and multiport RATS found no significant difference in the number of evaluated LNs between the two groups (18). In our cohort, nodal retrieval was comparable between the two groups, indicating that reducing the number of ports did not compromise the adequacy of nodal dissection. Accordingly, early oncologic outcomes appear to be preserved with a uniport approach. This may be explained by preservation of the robotic platform's technical advantages despite port reduction. Although this study did not include a direct comparison with VATS, the preserved nodal yield supports the oncologic feasibility of uniport RATS within the robotic platform. Nevertheless, given the limited evidence regarding long-term oncologic outcomes comparing uniport and multiport RATS, further studies are warranted to confirm their long-term efficacy.

Some limitations inherent to this study should be recognized. First, this analysis was conducted retrospectively at a single institution and involved a limited number of patients, which may introduce selection bias. Furthermore, because the operations were performed by a single surgeon, the results may not be directly generalizable to other settings. Although uniport RATS is currently the standard approach in our practice, the choice of surgical technique during the study period was at the surgeon's discretion. Second, because this study included the initial experience with uniport RATS beginning from the surgeon's first robotic pulmonary resection, perioperative outcomes were likely influenced by the learning curve. As uniport RATS was introduced in a stepwise manner, case allocation was not random and may have been influenced by technical complexity, particularly during the early adoption period; patients with extremely large tumors or severe adhesions may have been selectively avoided. Although patients who received neoadjuvant treatment were excluded and measured baseline characteristics were balanced using IPTW, residual confounding related to unmeasured surgical complexity, such as adhesions, anthracotic LNs, and hilar difficulty, cannot be completely excluded. Therefore, the present findings should be interpreted within the context of a structured transition strategy rather than a direct randomized comparison. In addition, some imbalance in the distribution of individual lobar locations remained after IPTW adjustment. Because tumor location may influence operative complexity and postoperative pain, residual confounding related to tumor location cannot be completely excluded. Third, the number of ports was gradually reduced from a four-port approach to a uniport technique as experience accumulated, resulting in heterogeneity in port configuration within the multiport group. This variability may have affected operative time, postoperative pain, and complication rates, and should be considered when interpreting the findings. Accordingly, the present results should be understood as reflecting a structured transition strategy rather than a direct head-to-head comparison under unrestricted case selection. Fourth, unlike previous studies that utilized only the da Vinci Xi platform, this study employed both the da Vinci Xi and X systems owing to the situation in our institution. However, our surgical team had already accumulated substantial experience with the da Vinci X platform, and neither prolonged docking times nor major intraoperative robotic arm collisions were observed. Lastly, this study focused on comparing uniport and multiport RATS in terms of early outcomes to evaluate the safety and feasibility of uniport RATS. The long-term oncologic impact of this approach remains to be clarified through future research.

## 5 Conclusions

Within a structured transition pathway, uniport RATS achieved perioperative outcomes and early oncologic feasibility comparable to those of multiport RATS, supporting its safety and technical applicability.

## Data Availability

The original contributions presented in the study are included in the article/Supplementary Material, further inquiries can be directed to the corresponding author/s.
